# Multifunctional Colloidal Quantum Dots-Based Light-Emitting Devices for On-Chip Integration

**DOI:** 10.3390/nano15181422

**Published:** 2025-09-16

**Authors:** Ruoyang Li, Jie Zhao, Yifei Qiao, Xiaoyan Liu, Shiliang Mei

**Affiliations:** Institute for Electric Light Sources, College of Intelligent Robotics and Advanced Manufacturing, Fudan University, Shanghai 200433, China; 22307130180@m.fudan.edu.cn (R.L.); 23210720135@m.fudan.edu.cn (J.Z.); 24210720028@m.fudan.edu.cn (Y.Q.); 22307130410@m.fudan.edu.cn (X.L.)

**Keywords:** colloidal quantum dots, quantum confinement, multifunctional light-emitting devices, on-chip integration

## Abstract

Colloidal quantum dots (CQDs) have attracted significant attention in optoelectronics due to their size-tunable bandgap, high photoluminescence quantum yield, and solution processability, which enable integration into compact and energy-efficient systems. This review consolidates recent progress in multifunctional CQD-based light-emitting devices and on-chip integration strategies. This review systematically examines fundamental CQD properties (quantum confinement, carrier dynamics, and core–shell heterostructures), key synthesis methods including hot injection, ligand-assisted reprecipitation, and microfluidic flow synthesis, and device innovations such as light-emitting field-effect transistors, light-emitting solar cells, and light-emitting memristors, alongside on-chip components including ongoing electrically pumped lasers and photodetectors. This review concludes that synergies in material engineering, device design, and system innovation are pivotal for next-generation optoelectronics, though challenges such as environmental instability, Auger recombination, and CMOS compatibility require future breakthroughs in atomic-layer deposition, 3D heterostructures, and data-driven optimization.

## 1. Introduction

Colloidal quantum dots (CQDs), a revolutionary class of nanomaterials (1~10 nm), have attracted significant attention in the field of optoelectronics over the past few decades [1,2,3,4,5,6]. This interest is primarily due to their size-dependent tunable bandgap, high photoluminescence quantum yield, solution processability, and compatibility with diverse substrates. These intrinsic properties make CQDs a unique platform for integrating light emission with both electronic and photonic functions, which is particularly valuable in addressing the growing demand for smarter, more compact, and energy-efficient systems in modern electronics.

With these exceptional properties, CQD-based multifunctional light-emitting devices, such as light-emitting field-effect transistors (LEFETs), light-emitting solar cells (LESCs), and light-emitting memristors (LEMs), are emerging as promising solutions. These devices can simultaneously integrate light emission with information processing [7,8,9], energy harvesting [10,11,12], and memory storage [13,14,15], thereby reducing the demand for complex external circuits required by traditional single-function emitters.

Furthermore, on-chip photonics has become another research frontier [16,17,18,19,20]. Traditional on-chip integration relies on heterogeneous integration of II–III–V lasers and detectors with silicon platforms, suffering from lattice mismatch and wavelength mismatch [21,22]. In contrast, CQDs can address these limitations by leveraging their solution processability and compatibility with existing CMOS technology, while their size-tunable optical properties allow precise spectral control for CQD-based lasers and detectors. This intrinsic material compatibility not only minimizes integration losses but also paves the way for scalable and high-performance photonic circuits [23], which is essential for next-generation optoelectronic technologies.

Despite significant advancements in CQD material development and their application in complex device systems, there remains a lack in comprehensive reviews that consolidate recent progress in multifunctional CQD-based light-emitting devices and on-chip integration strategies. This review aims to fill this gap by providing a holistic perspective on both device functionalities and integration approaches, thus offering a comprehensive understanding of the current state and future potential of CQD-based optoelectronics.

In this review, the fundamental properties of CQDs are first clarified, focusing on quantum confinement effects, carrier dynamics and core–shell heterostructures. Then, key synthesis strategies, including hot injection, ligand-assisted reprecipitation, and microfluidic flow synthesis, are systematically reviewed. Most importantly, the review delves into the diverse landscape of multifunctional light-emitting devices, such as light-emitting transistors, solar cells, and memristors followed by an in-depth analysis of on-chip integration strategies with emphasis on 2 critical components: CQD lasers as on-chip light sources and CQD detectors as high-sensitivity light receivers. Finally, the review concludes with an analysis of the current challenges and future opportunities in material engineering, device design, and system integration, guiding the development of next-generation CQD-based optoelectronic systems.

## 2. Properties of Colloidal Quantum Dots

CQDs exhibit distinct electronic and optical properties due to the quantum confinement effect, which becomes significant when their dimensions approach the exciton Bohr radius [24,25]. These properties are highly tunable and are critical in determining the performance of CQDs in a variety of optoelectronic devices. This section provides an in-depth examination of the fundamental properties of CQDs, including quantum confinement, carrier dynamics, and core–shell heterostructures, all of which play vital roles in their potential for integration into next-generation optoelectronic systems.

### 2.1. Quantum Confinement

**Figure 1:**
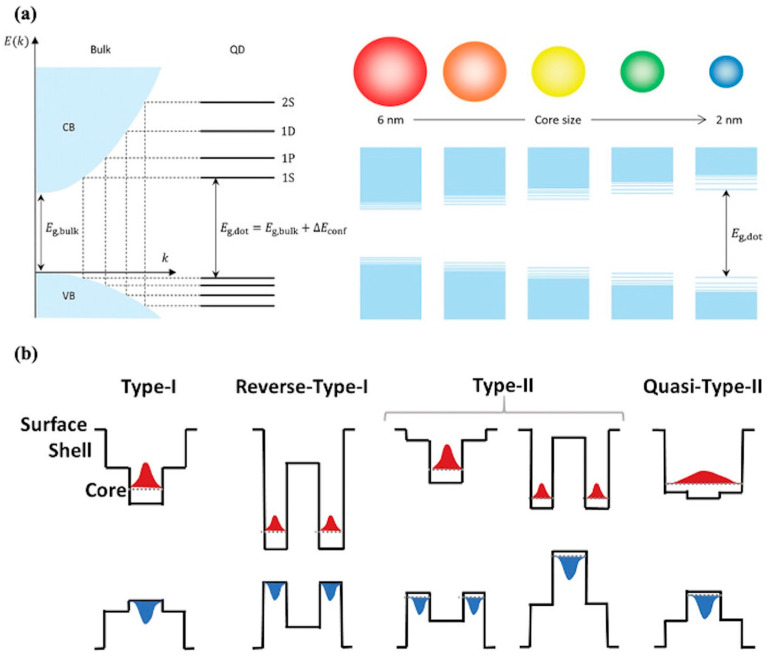
Schematic diagrams of fundamental properties of CQDs. (**a**) Quantum confinement induces a discrete, atom-like density of states, distinct from the continuous band structure of bulk semiconductors. This size-dependent electronic structure leads to tunable bandgaps, where smaller QDs exhibit larger bandgap, resulting in a blue shift of absorption and emission spectra [26]. (**b**) Core–shell heterostructures are classified into Type I and quasi-Type II based on the relative alignment of conduction band minima and valence band maxima, where Type I confines both electrons and holes within the core, while quasi-Type II allows partial wavefunction delocalization into the shell [27].

The quantum confinement effect is the key characteristic of CQDs. When the size of a semiconductor material is reduced to the range of exciton Bohr radius, the electronic states are confined within the nanoparticle, leading to discrete energy levels. In bulk materials, the energy levels form continuous bands; however, in QDs, the electron energy levels become quantized. The confinement of charge carriers in all three spatial dimensions alters the density of states (DoS), as depicted in Figure 1a. The relationship between the size of the CQD and its emission wavelength is governed by the equation for the quantum confinement energy:

(1) $$E_{\mathrm{conf}}\;=\;\frac{\pi^{2}\hbar^{2}}{2m^{*}d^{2}}$$

where $E_{\mathrm{conf}}$ is the confinement energy, ℏ is the reduced Planck’s constant, m* is the effective mass of the charge carriers, and d is the diameter of the quantum dot.

As the CQD size decreases, the bandgap between these energy levels increases, leading to a wider bandgap. This size-dependent bandgap allows for the precise tuning of optical properties such as absorption and emission wavelengths, which can be tailored by adjusting the size of the quantum dot.

### 2.2. Carrier Dynamics

Carrier dynamics in CQDs are essential for determining the performance of CQD-based optoelectronic devices, especially those that rely on the generation, recombination, and transport of charge carriers [26,28,29]. Several processes influence carrier behavior in CQDs, including single-exciton generation and decay, biexciton and multiexciton generation, optical gain, and the Auger process.

Single-Exciton Generation and Decay: This process involves the creation of an electron–hole pair (exciton), which can recombine radiatively, releasing energy as light. Efficient radiative recombination is essential for applications like light-emitting diodes (LEDs) and lasers, where high quantum efficiency is required [30,31,32,33,34,35].

Biexciton and Multiexciton Generation: Multiple excitons can be generated in a single CQD under high excitation, affecting the material’s optical properties [36,37]. While biexciton generation can enhance light emission, it may also introduce nonradiative recombination paths, reducing overall efficiency.

Optical Gain: Optical gain in CQDs arises when the number of excitons in an excited state exceeds that in the ground state, leading to a condition known as population inversion. In this state, stimulated emission dominates over absorption, amplifying incoming photons. This process is the foundation for lasers and optical amplifiers. The optical gain depends on factors such as CQD size, material composition, and surface engineering, all of which influence the exciton dynamics and the threshold for stimulated emission [38,39].

Auger Process: The Auger process is a nonradiative recombination mechanism where the energy released by an electron–hole recombination is transferred to a third carrier (electron or hole), which is then excited to a higher energy state. This process occurs when the interaction between charge carriers is strong, typically in smaller CQDs, and is detrimental to light emission efficiency. The Auger process competes with radiative recombination, reducing the overall quantum yield and limiting the performance of CQD-based light-emitting devices [40,41,42,43].

Controlling these carrier dynamics through factors like CQD size, composition, and surface passivation is crucial for optimizing CQD-based optoelectronic devices, ensuring maximum performance in applications such as light emission and energy harvesting.

### 2.3. Core–Shell Heterostructures

Core–shell heterostructures have been widely employed to enhance the optical and electronic properties of CQDs by passivating surface defects and improving carrier confinement. These structures consist of a semiconductor core material (e.g., CdSe, PbS, or InP) encapsulated by a shell material with a wider bandgap (e.g., ZnS, CdS, or ZnS). The shell material passivates the surface defects of the core [44,45] and reduces nonradiative recombination [46,47], leading to higher photoluminescence quantum yields (PLQY) and improved device stability.

The carrier dynamics and optical properties of core–shell CQDs are further modulated by the alignment of the conduction band and valence band edges between the core and shell. As shown in Figure 1b, core–shell structures can be categorized as follows:

Type I: Both the electron and hole are confined within the core, leading to efficient radiative recombination [48]. This configuration is ideal for light-emitting applications where high quantum efficiency is desired.

Type II: The electron and hole are spatially separated between the core and shell, which can result in longer carrier lifetimes and lower recombination rates. This configuration is useful for applications such as solar cells [27,44,49] and photodetectors [50,51], where charge separation is beneficial.

Quasi-Type II: The electron is primarily confined in the shell, while the hole remains in the core. This intermediate configuration provides a balance between carrier confinement and spatial separation, offering potential advantages in both light emission and charge separation.

To further optimize performance, multi-shell designs are often employed, where successive shells with tailored bandgaps minimize lattice mismatch between layers. Such engineering not only refines optical properties [52] but also enhances resistance to environmental stressors like oxidation and moisture [53], which is essential for their commercialization in optoelectronic devices.

## 3. Synthesis of Colloidal Quantum Dots

### 3.1. Hot Injection

Hot injection is the most typical method for synthesizing CQDs. Ever since Murray et al. first reported this method for II–VI semiconductor CQDs (e.g., CdSe) in 1993 [54], this method has since been adapted for diverse systems (e.g., lead halide perovskites, II–III–V semiconductors, and alloyed CQDs).

**Figure 2:**
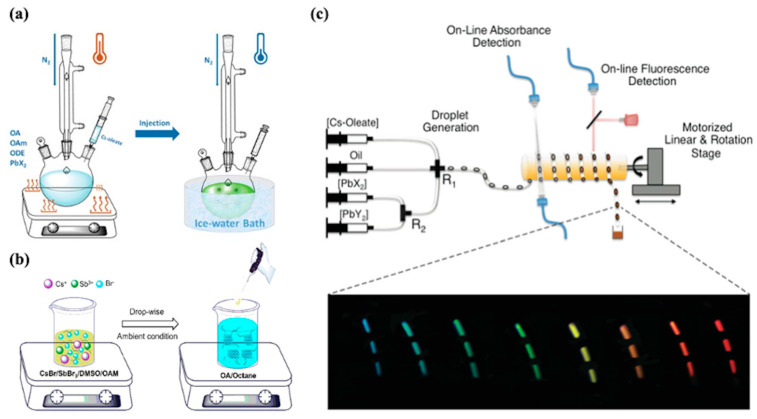
Schematic illustration of typical CQD synthesis methods including hot injection, ligand-assisted reprecipitation and Microfluidic Flow Synthesis. (**a**) The typical hot injection synthesis for metal-halide perovskite QDs [47]. (**b**) The modified LARP process for Cs_3_Sb_2_Br_9_ inorganic perovskite quantum dot synthesis [55]. (**c**) A droplet-based microfluidic platform integrates with online absorbance and fluorescence detection for the synthesis and real-time characterization of CsPbX_3_ perovskite nanocrystals [56].

The basic process of hot injection is illustrated in Figure 2a: (i) Under an inert atmosphere (e.g., nitrogen), precursors (e.g., PbX_2_, X = Cl, Br, and I) and coordinating ligands (e.g., oleic acid (OA) and oleylamine (OAm)) are mixed and dissolved in a solvent (e.g., octadecene, ODE), forming stable metal–ligand complexes upon heating to certain temperature. (ii) Another precursor (e.g., Cs-oleate) is rapidly injected into the above hot mixture. This abrupt injection induces an immediate surge in monomer supersaturation, triggering synchronous burst nucleation to form uniform nuclei. After a short size-focusing period (5–20 s), the reaction is quenched via an ice-water bath to terminate particle growth, followed by purification using antisolvents (e.g., methyl acetate) to remove excess ligands and precipitate the CQDs.

The size, morphology and optical properties of CQDs are highly sensitive to small changes in experiment parameters, such as reaction temperature [57,58], reaction solvents [59] and precursor concentration [60,61]. By gaining deeper insights into the nucleation and growth mechanism, along with the precise control of synthesis parameters, the hot injection method has shown significant advantages in producing high-quality QDs. However, the method is energy intensive, requires strict inert-gas environment, and relies on high-cost laboratory setups, which pose challenges for scalability and cost-effectiveness. Furthermore, the use of Cd- or Pb-containing precursors raises toxicity and environmental concerns, limiting its manufacturability for consumer-oriented applications [35,47,51].

In addition, the elevated temperatures inherent to hot injection can trigger side reactions between ligands and precursors (e.g., amidation of OA and OAm), leading to halide vacancies and lattice disorder. Such intrinsic defects act as nonradiative recombination centers, shortening carrier lifetimes and accelerating device degradation. Suppressing these side reactions during synthesis can yield CQDs with intrinsically lower defect densities, thereby enhancing charge transport, improving luminescence efficiency in LEDs, and boosting operational stability in solar cells.

### 3.2. Ligand-Assisted Reprecipitation

Ligand-assisted reprecipitation (LARP) has emerged as a transformative synthesis method for CQDs, particularly metal halide perovskites, enabling room-temperature, solution-processable fabrication with exceptional scalability.

LARP relies on the solvent polarity-induced supersaturation to drive nucleation and growth of CQDs [55], as schematically illustrated in Figure 2b. The process involves two key stages: (i) Under ambient conditions, metal halide precursors (e.g., SbBr3 and CsBr) and coordinating ligands (e.g., OAm) are dissolved in a polar solvent (e.g., dimethyl sulfoxide (DMSO) and N,N-dimethylformamide (DMF)) to form a clear precursor solution. The ligands coordinate with metal ions to stabilize the ionic complexes at room temperature. (ii) The precursor solution is then dropped into a nonpolar solvent (e.g., octane mixed with OA) under vigorous stirring. This sudden introduction into a poor solvent induces an immediate reduction in solubility, leading to supersaturation of the ionic species and triggering nucleation and growth of CQDs. After a short reaction period, the product is purified via centrifugation using antisolvents (e.g., acetone and octane) to remove excess ligands and residual solvents, yielding stable QDs in the nonpolar solvent phase.

Notably, unlike hot injection, LARP does not decouple nucleation and growth kinetically—both processes overlap due to ultrafast reaction rates [62]. Thus, the initial nucleation event (governed by solvent mixing dynamics) dictates the final size and shape of CQDs. High-throughput studies reveal that ligand diffusion at the polar/nonpolar interface is critical: long-chain ligands (e.g., OA/OAm) form more rigid reverse micelles, constraining growth to yield monodisperse cubic CQDs, whereas short-chain ligands (e.g., octanoic acid and octylamine) result in broader size distributions due to weaker van der Waals interactions [63,64,65]. Additionally, insulating ligands (e.g., OA/OAm) hinder carrier transport and reduce device stability. Post-synthetic ligand exchange—particularly with short-chain or π-conjugated ligands—can shorten interparticle spacing, enhance electronic coupling, and passivate trap states. Such surface reconstruction directly translates into higher photovoltaic efficiency, improved electroluminescence, and better resistance to environmental degradation.

Although the overlapping nucleation and growth stages result in lower crystallinity compared to hot injection, increasing surface defects and nonradiative recombination. Its room-temperature operation, minimal energy requirements, and compatibility with ambient conditions address key limitations of high-temperature methods like hot injection, making it amenable to large-scale synthesis.

### 3.3. Microfluidic Flow Synthesis

Unlike batch methods such as hot injection and ligand-assisted reprecipitation, microfluidic systems leverage microscale channels to manipulate fluid dynamics, mass/heat transfer, and reaction parameters, offering unique advantages for regulating and FastMapping of nucleation and growth kinetics [66,67,68].

The droplet-based microfluidic synthesis of CQDs, as exemplified by the CsPbX_3_ perovskite system in Figure 2c [56], proceeds through four stages: (i) Precursor solutions—including lead halides (e.g., PbX_2_ and PbY_2_), cesium oleate (Cs-oleate), and coordinating ligands (e.g., OA and OAm) dissolved in organic solvents (e.g., octadecene)—are separately loaded into precision syringe pumps. These pumps deliver the precursors into microchannels at controlled flow rates, which allows continuous adjustment of molar ratios (e.g., Pb:Cs ratio R_1_ and halide ratio R_2_). (ii) The precursor streams converge at a T-junction or cross-mixing junction, where they are rapidly mixed and segmented into discrete droplets by an immiscible carrier fluid (e.g., oil). These droplets act as isolated microreactors, preventing cross-contamination and ensuring uniform reaction conditions. (iii) The droplets flow through a temperature-controlled heating zone (120–200 °C), where nucleation and growth of CsPbX_3_ nanocrystals occur within a defined residence time (0.1–10 s), governed by the flow rate and channel length. Real-time monitoring via online absorbance and fluorescence spectroscopy tracks the evolution of nanocrystal size and optical properties. (iv) After exiting the heating zone, the reaction is naturally quenched as droplets cool. The product stream is collected, and nanocrystals are purified via centrifugation with antisolvents (e.g., methyl acetate) to remove excess ligands and residual solvents, yielding colloidal CsPbX_3_ QDs with tunable emission across the visible spectrum.

Nowadays, with the development of artificial intelligence for science, the reaction system becomes more intelligent and adaptive, enabling precise regulation of colloidal quantum dot synthesis. Integrating multi-modal sensors with microfluidic platforms allows real-time acquisition of critical parameters, such as precursor concentration, reaction temperature, residence time, and CQD optical properties (e.g., photoluminescence quantum yield and full width at half maximum). These high-dimensional datasets are then fed into machine learning models [69,70,71,72,73], which are trained to decode the complex relationships between synthesis parameters and CQD quality—for instance, predicting optimal Pb:Cs ratios (R_1_) and halide ratios (R_2_) for CsPbX_3_ PQDs to achieve narrow emission linewidths [74]. In the context of real-world implementation, microfluidics platform offers a promising route toward industry-compatible, high-throughput fabrication of CQDs with tunable properties. Combined with printing and scalable integration techniques, it represents one of the most viable pathways to manufacturable, CMOS-compatible CQD optoelectronics [75,76]. A comparative summary of the three synthesis strategies discussed in Section 3.1, Section 3.2 and Section 3.3, including their typical reaction conditions, advantages and disadvantages, is provided in Table 1 to facilitate a concise overview.

## 4. Multifunctional Light-Emitting CQD Devices

### 4.1. Light-Emitting Field-Effect Transistors

LEFETs integrate the electrical switching capabilities of field-effect transistors with the light emission properties of LEDs. This integration offers advantages such as higher integration density, reduced production costs, and energy efficiency [8,77,78]. LEFETs utilize ambipolar charge transport in a CQD semiconductor layer, enabling precise control of the emission position through gate voltage [7,79,80]. The tunable optical properties and high quantum efficiency of CQDs enhance LEFET performance, making them versatile for applications in optoelectronic systems, including material study [81,82,83], charge storage [84,85], light emission [86,87,88,89] and optical communication [90,91].

**Figure 3:**
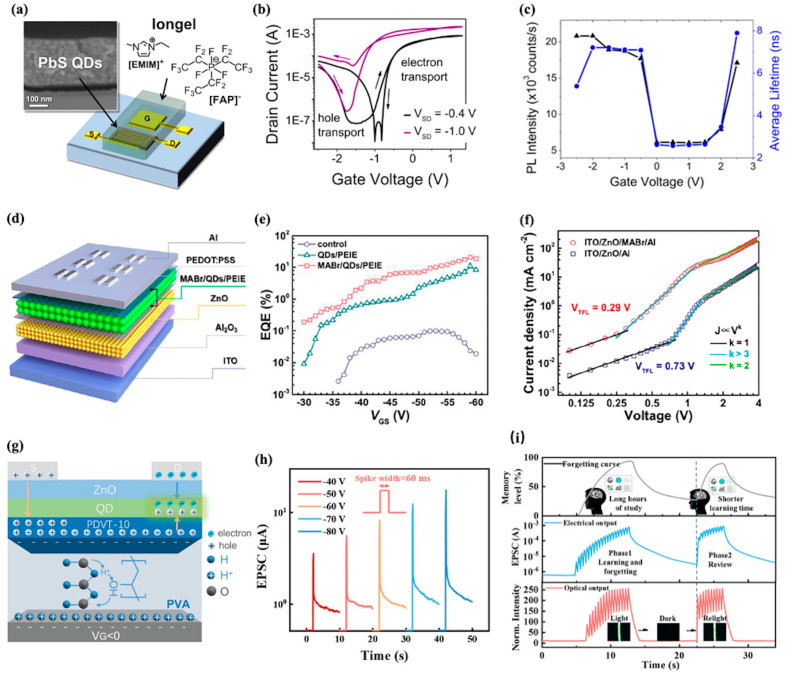
(**a**) Schematic illustration of the PbS QD LEFET with a side-gate geometry, comprising a PbS QD channel layer, source/drain electrodes, and an iongel electrolyte (incorporating [EMIM] [FAP] ionic liquid). (**b**) Transfer characteristics of an electrolyte-gated PbS QD FET. (**c**) Integrated PL intensity (black triangles) and average PL lifetime (blue circles) of PbS QDs versus gate voltage [92]. (**d**) Schematic diagram of the QD LEFET structure. (**e**) EQE versus gate voltage (V_GS_) curves of QLEFETs with different structures. (**f**) Trap-filled space-charge-limited current J–V curves for electron-only ITO/ZnO/Al and ITO/ZnO/MABr/Al devices [93]. (**g**) Schematic of the QD LEST’s dual-function mechanism: under negative gate bias, proton migration in the PVA dielectric drives hole accumulation in the PDVT-10 channel while electrons injected through the ZnO layer recombine in the CdSe:ZnS QD film to produce both excitatory postsynaptic currents and transient electroluminescence. (**h**) Excitatory postsynaptic currents of the LEST triggered by presynaptic electrical spikes with varying amplitudes (−40 to −80 V, 60 ms duration). (**i**) Electrical and optical simulation of the Ebbinghaus forgetting curve [94].

For instance, in 2015, Schornbaum et al. pioneered the development of the first ambipolar, electrolyte-gated LEFET using PbS CQD film as shown in Figure 3a [92]. The design is pivotal for charge carrier management: the iongel forms nanometer-thin electric-double layers (EDLs) that enable efficient accumulation of high carrier densities (~10^13^ cm^−2^) via gate voltage (V_GS_), while the side-gate configuration facilitates independent modulation of electron/hole injection (from source/drain) alongside V_GS_, laying the foundation for precise control of electron–hole recombination. Figure 3b presents the transfer characteristics of this electrolyte-gated LEFET, showing a V-shaped profile that reflects balanced ambipolar transport (electron mobility μₑ = 0.04–0.06 cm^2^ V^−1^ s^−1^, hole mobility μₕ = 0.003–0.009 cm^2^ V^−1^ s^−1^). This balanced charge injection mitigates nonradiative losses (e.g., trap-induced recombination) that often plague conventional CQD devices, analogous to mitigating Auger recombination via excess carrier control. Figure 3c further correlates gate voltage-dependent PL intensity and average emission lifetime of PbS QDs (d = 4.6 nm). It exhibits a ~10-fold PL enhancement and lifetime extension (2–3 ns to 1–12 ns) when V_G_ is tuned to ±2 V (electron/hole accumulation). This phenomenon confirms that high carrier densities (facilitated by EDLs) deactivate nonradiative trap states, directly underpinning the device’s efficiency performance, while this study reports modest external quantum efficiencies (EQE ~0.002%), the trend of efficiency improvement with current density (unlike conventional QD LEDs with efficiency roll-off) provides a solution to address the limitation of traditional light-emitting devices. 

Subsequently, in 2018, Shulga et al. further advanced the development of PbS quantum dot LEFETs by demonstrating the use of solid-state gating [83]. The team utilized PbS QDs treated with tetrabutylammonium iodide (TBAI), enabling high quantum efficiency of over 1% at low temperatures. By analyzing charge transport at various temperatures, they highlighted the role of hole trap states in limiting the performance at higher temperatures, with notable improvements in electroluminescence efficiency at lower temperatures. Both studies underscore CQDs as robust emitters for LEFETs, with ligand engineering (MPA or TBAI) and trap state management (via high carrier densities or low temperatures) being critical to balancing charge transport and radiative recombination.

In recent years, great breakthroughs have been achieved in LEFET performance, building on these foundational studies. These breakthroughs have been particularly notable in achieving high EQE [95,96,97] and expanding the technology’s potential in optical communication [98,99].

He et al. constructed a quantum-dot hybrid light-emitting field-effect transistor (QD-HLET) via a solution-processed strategy [100]: adopting a bottom-gate, top-emitting, asymmetric nonplanar source/drain architecture, its functional layer stack included an InScO/ZnO-nanoparticle heterojunction (high-mobility electron channel), a colloidal core–shell QD layer (emitting layer), and a TCTA organic hole transport layer, with key layers (InScO, ZnO nanoparticles, QDs) fabricated by spin-coating and TCTA/electrodes by vacuum thermal evaporation. This QD-HLET exhibited exceptional performance: 3.1 cm^2^ V^−1^ s^−1^ field-effect mobility, 145,000 cd m^−2^ maximum brightness, and a peak EQE of 22.8% (surpassing state-of-the-art LETs and top-emitting QLEDs), with EQE > 20% over 0.3–193 mA cm^−2^, low roll-off (15.7% EQE at 1000 mA cm^−2^), and ~153,000 h T50 lifetime at 100 cd m^−2^, significantly outperforming the equivalent top-emitting QLED which only achieves 28,000 h under the same conditions.

In 2022, Kong et al. constructed a dielectric–QDs–dielectric (DQD) sandwich structure in LEFETs to optimize charge transport and exciton recombination processes [93]. As depicted in Figure 3d, by incorporating ZnO nanoparticles as the electron transport layer (ETL) and integrating polyethylenimine ethoxylated (PEIE) and methylammonium bromide (MABr) dielectric layers around the QDs, they achieved significant improvements in device performance. Figure 3e reveals the EQE of the DQD-structured devices reached up to 21%, a remarkable enhancement compared to previous designs. The devices also exhibited high luminance, with a maximum of 13,320 cd m^−2^, highlighting the effectiveness of the DQD configuration in boosting light-emitting performance. Moreover, Figure 3f shows trap-state density in the QD films. The introduction of the MABr layer significantly reduced the trap-state density from 2.1 × 10^17^ cm^−3^ for ZnO film to 6.3 × 10^12^ cm^−3^ for ZnO/MABr film. This reduction in trap states is crucial for improving charge transport and reducing charge carrier loss at the interface, leading to enhanced electron current and overall device efficiency.

Building on the progress in enhancing LEFETs’ EQE, their evolution toward optoelectronic interconnection has expanded into light communication, with the quantum dot light-emitting synaptic transistor (LEST) emerging as a key advancement. This device integrates light emission and synaptic functionality, enabling parallel photoelectric signal transmission to address wire crosstalk in large-scale networks. As shown in Figure 3g, its mechanism relies on proton migration in the PVA dielectric, modulating hole accumulation in the PDVT-10 channel and electron–hole recombination in QDs [94]. This dual regulation of conductance and luminescence underpins its communication capability, leveraging QDs’ high color purity and synaptic plasticity beyond traditional LEFETs’ focus on luminous efficiency. Figure 3h demonstrates its high-fidelity signal encoding potential: under varying presynaptic pulse amplitudes (−40 to −80 V), excitatory postsynaptic current (EPSC) grades from 3.57 to 17.6 μA with synchronized electroluminescence changes, enabling multi-level information encoding into optical signals for high-bandwidth communication. Meanwhile, Figure 3i highlights suitability for time-dependent processing, as the device reproduces the Ebbinghaus forgetting curve via electrical conductance decay and luminescence fading, enabling spatiotemporal information encoding critical for parallel transmission. By integrating memory with emission, LESTs advance LEFETs from efficiency-optimized emitters to multifunctional transceivers, redefining next-generation light communication.

### 4.2. Light-Emitting Solar Cells

As highlighted in previous reviews, the dual-mode operation of CQD-based LESCs, which harvest solar energy by day and emit light by night, addresses critical needs in self-powered lighting, smart architecture, and portable electronics [101,102,103,104]. Key to their performance is the rational engineering of CQD surfaces and interfaces to balance charge extraction (for power conversion) and radiative recombination (for light emission), as underscored by advancements in defect passivation [105,106,107,108], ligand engineering [109,110,111,112], and energy-level alignment strategies [113,114,115]. These foundational insights, rooted in studies of perovskite and hybrid QD systems, set the stage for exploring the working principles, material optimizations, and performance benchmarks of CQD-based LESCs.

**Figure 4:**
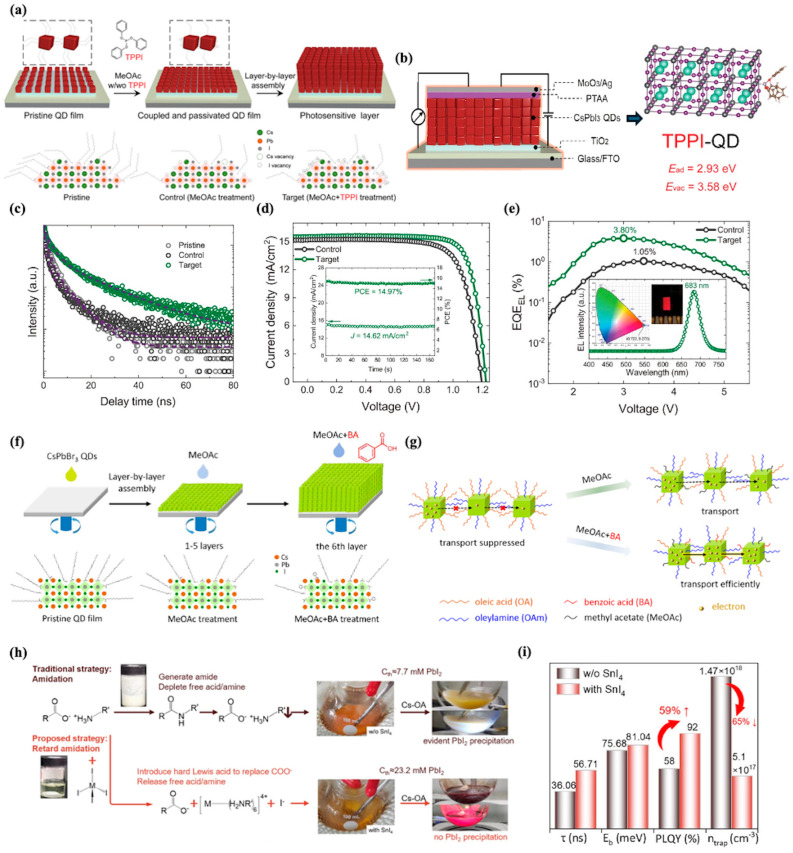
(**a**) Top: Schematic illustration of the layer-by-layer assembly process for CsPbI_3_ QD photosensitive layers. Down: Schematic of CsPbI_3_ QD surface states: pristine QDs with native ligands, control QDs after MeOAc treatment (exhibiting surface vacancies and residual long-chain ligands), and target QDs after MeOAc + TPPI treatment (with TPPI passivating vacancies and bonding to undercoordinated ions). (**b**) Left: Device structure illustration. Right: Density functional theory results showing adsorption energies (E_ad_) and surface iodine vacancy formation energies (E_vac_) for TPPI ligands on CsPbI_3_ QDs. (**c**) TRPL spectra of pristine, control (MeOAc-treated), and target (MeOAc + TPPI-treated) CsPbI_3_ QD films (excitation wavelength: 455 nm). (**d**) Current density–voltage curves of control and target CsPbI_3_ QD solar cells under AM1.5G illumination. (**e**) Electroluminescent external quantum efficiency curves of control and target CsPbI_3_ QD devices [116]. (**f**) Schematic illustration of the layer-by-layer deposition process for CsPbBr_3_ quantum dot films under ambient conditions. (**g**) Schematic diagram depicting the effect of ligand treatments on CsPbBr_3_ quantum dot films [117]. (**h**) Schematic illustration comparing the reaction pathways in Pb precursors with and without SnI_4_. (**i**) Summary of optical properties of CsPbI_3_ PQDs with and without SnI_4_ treatment [118].

In 2021, Wang et al. achieved a pivotal breakthrough in CsPbI_3_ perovskite QD-based electroluminescent solar cells through the strategic introduction of triphenyl phosphite (TPPI) as a tailored ligand via a solid-state ligand exchange process, thereby synergistically enhancing both photovoltaic (PV) and electroluminescent (EL) functionalities [116]. The fabrication of the QD photoactive layer relies on a layer-by-layer assembly process, as schematically illustrated in Figure 4a: CsPbI_3_ QD films are deposited sequentially, with each layer subjected to post-treatment using a methyl acetate (MeOAc) solution containing TPPI to facilitate ligand exchange, effectively replacing the native long-chain oleate/oleylammonium ligands. This surface modification profoundly alters the QD surface chemistry. TPPI-treated QDs exhibit markedly reduced Cs and I vacancies compared to control films treated with neat MeOAc, underscoring the ligand’s role in defect passivation. The device architecture, illustrated in Figure 4b, adopts a planar n-i-p configuration (glass/FTO/TiO_2_/CsPbI_3_ QDs/PTAA/MoO_3_/Ag), where the TPPI-engineered QD layer serves as the dual-functional core, enabling both light harvesting and emission. The mechanistic basis for this performance enhancement is validated by density functional theory (DFT) calculations, which reveal that TPPI binds strongly to the QD surface with an adsorption energy (E_ad_) of 2.93 eV—far exceeding that of conventional oleate ligands (0.53 eV)—and increases the formation energy of surface I vacancies to 3.58 eV, thereby suppressing nonradiative recombination pathways.

This rational design yields exceptional device performance: Time-resolved photoluminescence (TRPL) measurements shown in Figure 4c reveal that the TPPI-treated CsPbI_3_ QD film exhibits a prolonged average carrier lifetime of 6.5 ns, which is significantly longer than that of the control film treated with neat MeOAc (36.06 ns for pristine, shorter for control), directly indicating the effective suppression of trap-mediated nonradiative recombination. In photovoltaic mode, the current density–voltage (J–V) characteristics demonstrate that the optimized TPPI-treated device achieves a power conversion efficiency (PCE) of 15.21% (14.97% at the maximum power point), accompanied by an open-circuit voltage (V_oc_) of 1.23 V, a short-circuit current density (J_sc_) of 15.54 mA cm^−2^, and a fill factor (FF) of 79.45%. This performance notably outperforms the control device, which yields a maximum PCE of 13.55% with a V_oc_ of 1.20 V, J_sc_ of 15.15 mA cm^−2^, and FF of 74.49%. Concurrently, in electroluminescent mode presented in Figure 4e, the TPPI-treated device exhibits a peak EQEel of 3.80% at an applied voltage of 3.0 V, with sharp red emission centered at 683 nm (full width at half maximum ≈30 nm). This represents a more than threefold enhancement compared to the control device, which shows a maximum EQEₑₗ of 1.05%, highlighting the critical role of TPPI ligand engineering in balancing charge transport and radiative recombination.

Extending the ligand engineering strategy to broader perovskite QD systems, Wang et al. reported a complementary approach for CsPbBr_3_ QD-based LESCs, leveraging aromatic carboxylic acid ligands to enhance dual-function performance [117]. Similar to the layer-by-layer assembly process for CsPbI_3_ QDs, the fabrication of CsPbBr_3_ QD films employs sequential deposition followed by post-treatment, as schematically illustrated in Figure 4f: after depositing ~300 nm thick QD layers, methyl acetate (MeOAc) containing benzoic acid (BA) (MeOAc + BA) is used to replace native long-chain oleate/oleylamine ligands, a process that optimizes surface chemistry for both PV and EL modes. The key role of BA in improving device performance is visualized in Figure 4g: compared to MeOAc treatment alone, MeOAc + BA effectively removes insulating long-chain ligands, shortens inter-QD distances, and utilizes the π-conjugated benzene ring of BA to enhance electronic coupling between QDs. This dual action suppresses trap-assisted nonradiative recombination and boosts charge transport—critical for balancing light absorption (PV mode) and radiative emission (EL mode). Consequently, the champion CsPbBr_3_ QD LESC achieves a PCE of 5.46% in PV mode, with a J_sc_ of 4.84 mA cm^−2^, FF of 71.0%, and V_oc_ of 1.59 V, while functioning as a green LED with a maximum luminance of 584 cd m^−2^ in EL mode. This work underscores the versatility of ligand engineering in tailoring perovskite QDs for multifunctional optoelectronics, complementing the advancements in CsPbI_3_ systems with strategies optimized for wide-bandgap CsPbBr_3_.

Beyond ligand engineering, defect passivation during the synthesis of PQDs represents another critical strategy to enhance the performance of multifunctional optoelectronic devices. In 2025, Su et al. proposed an amidation-retarded synthesis approach to mitigate defect formation in CsPbI_3_ PQDs, addressing a fundamental limitation in conventional hot-injection methods: unavoidable PbX_2_ precipitation induced by ligand amidation at high temperatures, which generates abundant internal and surface defects [118]. As schematically illustrated in Figure 4h, traditional synthesis suffers from intense amidation between OA and OAm ligands under elevated temperatures, depleting free ligands and causing PbI_2_ precipitation—this leads to irregular lead–halide octahedra and disordered crystal growth, introducing a high density of traps. In contrast, the proposed strategy introduces covalent metal halides (e.g., SnI_4_) to interrupt amidation by reacting with deprotonated OA/protonated OAm, releasing free acids/amines to coordinate with PbX_2_ and promote the formation of regular lead–halide octahedra, thereby suppressing defect generation at the nucleation stage. The effectiveness of this defect passivation strategy is quantified in the optical property summary in Figure 4i, which reveals that SnI_4_-treated CsPbI_3_ PQDs exhibit a 65% reduction in defect density (from 1.47 × 10^18^ cm^−3^ to 5.1 × 10^17^ cm^−3^), a 57% increase in average carrier lifetime (from 36.06 ns to 56.71 ns), and a 59% enhancement in photoluminescence quantum yield (PLQY, from 58% to 92%). Concurrently, the exciton binding energy increases from 75.68 meV to 81.04 meV, indicating stronger exciton confinement and suppressed nonradiative recombination.

### 4.3. Light-Emitting Memristors

Optoelectronic memristors that synergize optical and electrical functionalities have emerged as pivotal building blocks for next-generation neuromorphic computing and optoelectronic integrated systems, offering unique advantages such as high bandwidth, low crosstalk, and the ability to mimic biological visual perception and synaptic plasticity [13,15,119]. The quantum confinement effects of CQD enable precise engineering of light emission wavelengths, while their rich surface states and carrier dynamics provide versatile pathways for realizing resistive switching and light-modulated synaptic behaviors. These characteristics further support their application in constructing neural networks, highlighting their significance in advancing optoelectronic integrated systems [120,121,122,123,124].

**Figure 5:**
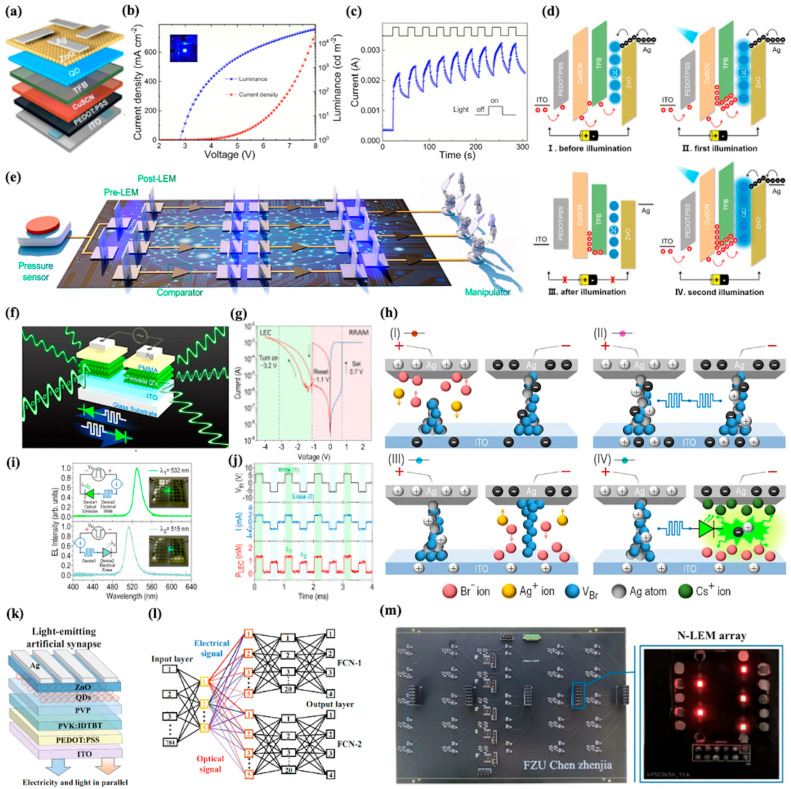
(**a**) Structure illustration of the LEM. (**b**) Current density–voltage–luminance (J–V–L) characteristics of the LEM. (**c**) Optical switching characteristics of the LEM under cyclical blue light stimulation, where the photocurrent gradually increases and saturates after nine cycles. (**d**) Schematic illustrations of hole trapping in the LEM. (**e**) Schematic diagram of the optoelectronic artificial efferent neural system, where external pressure signals detected by a pressure sensor are transmitted through optoelectronic synapses and electronic nerves to control manipulators intelligently [125]. (**f**) Schematic of the all-inorganic perovskite-based light-emitting memory composed of two identical Ag/CsPbBr_3_ QDs/ITO devices. (**g**) Current–voltage characteristics of a single CsPbBr_3_ QD-based device under dc bias sweeping (0 V → + 2 V → −4 V → 0 V). (**h**) Schematic illustrations of ion migration processes in the LEM. (**i**) Electroluminescence spectra of a two-color LEM with CsPbBr_3_ QDs of different sizes, emitting at 532 nm (green, write state) and 515 nm (aqua, erase state). (**j**) Time traces of current and optical power for the two-color LEM under 1 kHz alternating bias, demonstrating real-time distinction between write (higher power, 532 nm) and erase (lower power, 515 nm) states via optical power modulation [126]. (**k**) Structure diagram of the negative ultraviolet (UV) photoconductive light-emitting memristor with dual-output capabilities. (**l**) Schematic of two fully connected networks (FCN-1 and FCN-2) sharing an input layer and hidden layer 1, where FCN-1 uses electrical signals (postsynaptic current) and FCN-2 employs optical signals (postsynaptic brightness) for cross-layer transmission. (**m**) PCB hardware diagram of synaptic connections between hidden layers in FCN-1 and FCN-2, highlighting the position of the N-LEM array (for weight expression) and the corresponding photodiode array (for optical signal reception) [127].

In 2021, Zhu et al. reported the first CQD-based LEM with a layered ITO/PEDOT:PSS/CuSCN/TFB/CdSe/ZnS QDs/ZnO/Ag structure, as shown in Figure 5a [125]. This device integrated light emission, reception, and memristive functions; CdSe/ZnS QDs served as the emissive layer. Figure 5b exhibits the current density–voltage-luminance characteristics, revealing typical LED behavior. The device emitted blue light (452 nm) under forward bias, with luminance increasing from 10 to 10,334 cd m^−2^ as voltage rose from 3 to 6 V. Under cyclical blue light stimulation, the LEM exhibited stable resistive switching. Conductance increased by ~10 times after nine cycles (Figure 5c). This increase was attributed to hole trapping in the CuSCN layer, as elaborated by the working mechanisms illustrated in Figure 5d: (I) and (II) depict the initial hole transport and the first light stimulation process—before illumination, under voltage stimulation, holes transport from the anode through the PEDOT:PSS/CuSCN/TFB layers toward the CdSe/ZnS QD emissive layer with a certain efficiency; upon the first light stimulation, the TFB layer absorbs blue light from another LEM, leading to the separation of electron–hole pairs and generating photoinduced holes, which are injected into the QD layer to significantly enhance device current and promote blue emission, while part of the holes start migrating toward the CuSCN layer. (III) and (IV) illustrate the post-illumination state and the second light stimulation response—after the first illumination is removed, a portion of the photoinduced holes are trapped in the CuSCN layer due to its unique hole collection property; during the second light stimulation, the trapped holes in the CuSCN layer create a built-in electric field, effectively facilitating the transport of newly generated photoinduced holes from the TFB layer to the QD layer, resulting in a further increase in device current and a more pronounced synaptic response, thus realizing light-modulated synaptic plasticity. Leveraging these properties, the researchers constructed an optoelectronic artificial efferent neural system (Figure 5e). External pressure signals, detected by a sensor, trigger pre-LEM light emission. This modulates post-LEM resistance, which controls manipulators intelligently.

Yen et al. demonstrated an all-inorganic perovskite light-emitting memory device based on CsPbBr_3_ CQDs [126]. As illustrated in Figure 5f, this device consists of two serially connected Ag/CsPbBr_3_ QDs/ITO structures with a protective layer of poly(methyl methacrylate) (PMMA). This symmetric structure allows the devices to switch dynamically between resistive random-access memory (RRAM) and light-emitting electrochemical cell (LEC) functionalities via modulation of the bias polarity. Figure 5g exhibits the DC I–V characteristics, which confirm this dual functionality. Under positive bias, the device operates as an RRAM with set and reset voltages of ~0.7 V and ~−1.1 V, respectively. Under negative bias exceeding −3.2 V, the device transitions to an LEC with visible electroluminescence (EL) and exhibits distinct electrical hysteresis due to ion migration kinetics. The working mechanisms underlying this phenomenon involve field-induced ionic motions, as illustrated in Figure 5h. In regions (I) through (IV) of positive bias, Ag^+^ cations and Br^−^ vacancies migrate to form or reconstruct conductive filaments. (I) the initial formation of Ag filaments and Br^−^ vacancy channels in the left device, (II) both devices functioning as ON-state RRAMs, (III) the annihilation of filaments in the right device, and (IV) the formation of a p-i-n homojunction in the right device as an LEC, while the left device remains as an RRAM and emits light. This ion-mediated transition between resistive switching and light emission is the core functionality of the LEM. By engineering CsPbBr_3_ QDs of two different sizes, the LEM achieves dual-color emission (Figure 5i): 532 nm (green) from the larger QDs and 515 nm (aqua) from the smaller QDs. This leverages quantum confinement effects, visually distinguishing the “write” and “erase” states in real time. Figure 5j demonstrates fast switching at 1 kHz with synchronous current and optical power responses, validating parallel electrical readout via RRAM and optical transmission via LEC.

Building on their previous work in 2021, Chen et al. introduced a novel negative ultraviolet photoconductive light-emitting memristor (N-LEM) with dual-output capabilities, as depicted in Figure 5k [127]. The device structure, featuring an IDTBT/PVP/QDs interface, is an evolution from their previous CQD-based LEMs. This new interface design enables UV-modulated weight reset, a significant improvement over the previous model that relied on reverse voltage for weight adjustment, which was prone to device breakdown. The N-LEM’s dual-output characteristics, both in terms of electrical current and optical brightness, show a remarkable Pearson correlation coefficient of 0.999 (Figure 5l). This high correlation is a crucial enhancement compared to the single-modality output of the 2021 device. It allows for the equivalent cross-layer transmission of information through optical signals. To validate this, the researchers constructed two fully connected networks (FCNs) that share input and hidden layers. Figure 5m demonstrates that FCN-1 processes electrical outputs, while FCN-2 receives optical signals via photodiodes. The hardware circuit, composed of N-LEM arrays and customized PCBs, achieved classification accuracies of 91.6% for FCN-1 and 90.23% for FCN-2. These results are close to the ideal software accuracy of 94.95%, representing a substantial advancement from the relatively simple neural network demonstrations in their 2021 study. Moreover, the UV negative photoconductive effect in the N-LEM enables rapid weight reset, with a reset time ≤3.52% of the natural decay time. This efficiency is a significant leap forward, overcoming a major limitation of the earlier device. In combination with cross-layer transmission blocks (ClBlocks), the N-LEM facilitates the construction of 54-layer ultra-deep photoelectric neural networks (UPENN) with transfer learning capabilities, far exceeding the shallow network architectures achievable with their 2021 CQD-based LEMs.

### 4.4. On-Chip Integration

#### 4.4.1. Toward Electrically Pumped Lasers

Electrically pumped CQD lasers represent a promising candidate for on-chip light sources, merging the solution processability, size-tunable emission, and low-cost fabrication of CQDs with the practicality of electrical excitation—essential for compact, energy-efficient light sources in optical interconnects and quantum information processing [21,128,129]. Their realization, however, hinges on overcoming pivotal challenges: fast nonradiative Auger recombination of gain-active multicarrier states, inefficient charge injection under high current densities, and the difficulty of integrating high-quality optical cavities without compromising electrical performance [130,131,132,133]. Recent breakthroughs, driven by synergistic advances in material engineering (e.g., continuously graded core–shell heterostructures) and device design (e.g., current-focusing architectures and low-loss cavity integration), have substantially mitigated these barriers.

Lim et al. adopted the design of continuously graded QDs (cg-QDs) implemented with a CdSe/Cd_x_Zn_1−x_Se architecture [134]. The electronic structure incorporates a radially graded Cd_x_Zn_1−x_Se shell that smoothly modulates the confinement potentials for both electrons and holes. This special structural engineering suppresses nonradiative Auger recombination by minimizing abrupt interfacial transitions, thereby extending the lifetime of gain-active multicarrier states. It is a critical prerequisite for sustaining population inversion under electrical injection. The optical characteristics reveal a well-defined band-edge emission at ~2.02 eV with a narrow photoluminescence (PL) linewidth, accompanied by distinct absorption features corresponding to 1S and 1P transitions. The close overlap between the PL peak and the absorption edge, combined with the resolved splitting of the 1S heavy-hole and light-hole states (~35 meV), underscores precise control over quantum confinement and electronic states. This study lays a critical foundation for developing solution-processable, electrically driven colloidal QD lasers.

Building upon the advancements with cg-QDs, Hahm et al. proposed a new approach through the development of type (I + II) QDs, which integrates both spatially direct (type I) and indirect (type II) excitonic transitions [135]. The CdSe/ZnSe/CdS/ZnS structure enables hybrid direct/indirect biexcitons with trion-like Auger dynamics, where the Auger decay rate is suppressed to a single positive-trion pathway (rA,X_+_), significantly extending gain lifetimes (up to 3.0 ns) compared to cg-QDs. While electrical pumping remains experimentally unvalidated for this specific heterostructure, its band-engineered confinement potentials provide a critical design paradigm for suppressing Auger recombination in electrically driven quantum dot lasers.

**Figure 6:**
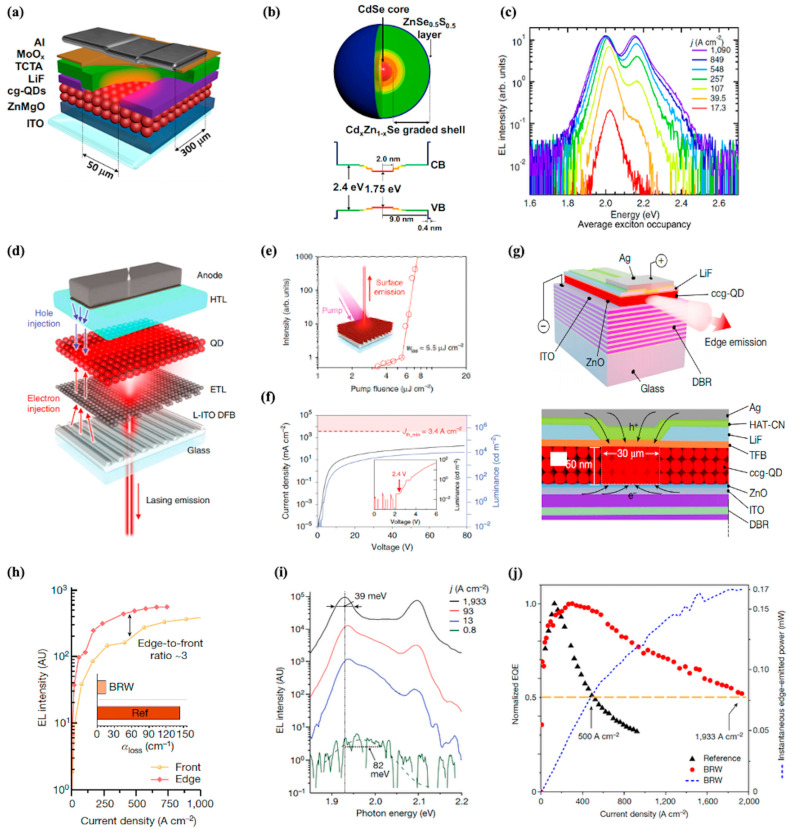
(**a**) Device structure of the current-focusing cg-QD LED. (**b**) Schematic illustration of the internal structure of continuously graded CdSe/Cd_x_Zn_1−x_Se/ZnSe_0.5_S_0.5_ QDs and their corresponding conduction band (CB) and valence band (VB) confinement potentials (**c**) EL spectra of the current-focusing LED under pulsed bias (1 μs, 100 Hz) as a function of current density, with the 1P band overcoming the 1S band at ultra-high current densities (>1000 A cm^−2^), indicating high exciton occupancies [136]. (**d**) Schematic of the proposed quantum dot laser diode (QLD) architecture, integrating a second-order distributed feedback (DFB) resonator into the low-index ITO (L-ITO) cathode of an inverted p-i-n QD-LED. (**e**) Surface emission intensity as a function of pump fluence for the L-ITO DFB/QD laser. (**f**) Current density–luminance–voltage (J–L–V) characteristics of the LED-like DFB device [137]. (**g**) Schematic of the BRW device structure. (**h**) Front/Edge EL intensity-current density for BRW device. (**i**) Edge-emitted EL spectra of the BRW device across current densities (0.8–1933 A cm^−2^). (**j**) J-dependent instantaneous edge-emitted power (dashed blue line) and external quantum efficiency (EQE, red circles) of the BRW device [138].

To achieve population inversion in CQD materials, the average exciton occupancy per quantum dot must exceed one [131]. Under electrical pumping conditions, this requires extremely high current densities, typically much higher than those used in conventional CQD LED devices. Jung et al. overcame the current-density bottleneck through a LiF-confined aperture (50 μm slit) combined with a 300 μm-wide Al anode, reducing the injection area to 0.015 mm^2^ (Figure 6a) [136]. This enabled record pulsed current densities of 1170 A cm^−2^ (τ_p_ = 1 μs, f_p_ = 100 Hz) while suppressing Joule heating to <70 °C. Like previous study, they adopted the design of continuously graded CdSe/Cd_x_Zn_1−x_Se/ZnSe_0.5_S_0.5_ QDs. The radially graded Cd_x_Zn_1−x_Se shell eliminates sharp discontinuities in the confinement potential, strongly suppressing nonradiative Auger recombination (biexciton lifetime ≈ 1.2 ns, 20-fold longer than standard CdSe QDs) and extending the stability of gain-active multiexciton states. The outer ZnSe_0.5_S_0.5_ layer further passivates surface defects, while the well-matched conduction band (CB, ~2.4 eV) and valence band (VB, ~1.75 eV) energies ensure efficient carrier trapping into the CdSe core (Figure 6b). As illustrated in Figure 6c, complete population inversion is achieved via a dominant 1P band (1P/1S intensity ratio = 1.24). This signals extreme excitonic occupancies of ~8 excitons per dot, which is sufficient to saturate the 2-fold degenerate 1S and 6-fold degenerate 1P electron shells. Such complete population inversion of both 1S and 1P transitions confirms the viability of cg-QDs for high-gain applications.

While sufficient injection current densities can induce strong optical gain in quantum dot materials, achieving amplified spontaneous emission (ASE) further requires the total optical gain in the device to exceed optical losses [132]. In this context, cavity integration is essential for reducing losses and achieving low-threshold electrically pumped CQD lasers. Studies on quantum dot lasing have investigated a range of cavity configurations, such as Fabry-Perot, microring resonators, distributed Bragg reflectors (DBR), and distributed feedback (DFB) gratings. In 2020, as presented in Figure 6d, Roh et al. engineered a 2nd-order DFB resonator onto a low-index ITO (L-ITO) cathode, merging the optical cavity with an LED-like stack to enhance mode confinement and minimize losses from conductive layers [137]. This architecture enables low-threshold lasing with a pump fluence of 5.5 μJ cm^−2^ (Figure 6e), where gain clearly surpasses losses, while its electroluminescence characteristics (Figure 6f) confirm stable electrical operation (~2.4 V turn-on voltage). This dual-function (lasing/EL) structures provide a promising platform for CQD laser diodes.

Notably, in 2023, Ahn et al. first achieved ASE from electrically pumped CQDs. Researchers engineered a compact version of CdSe/Cd_1−x_Zn_x_Se cg-QDs with thinner graded layer and a Bragg reflection waveguide (BRW) architecture that synergizes a DBR bottom mirror with a top Ag electrode (Figure 6g) [138]. This photonic structure reshapes the optical mode profile and enhances mode confinement in the compact cg-QD (ccg-QD) layer—evident from the strong edge emission (edge-to-front intensity ratio ~3) compared to reference devices (Figure 6h). Figure 6i demonstrates that as current density increases, sharp 1S and 1P ASE bands emerge with line narrowing from 82 to 39 meV, which confirms gains surpassing losses. As shown in Figure 6j, the BRW device achieves record output power (170 μW at 1933 A cm^−2^) and enhanced stability (>90% power retention after 2 h operation). Notably, the BRW architecture extends the efficiency droop onset to j_1/2_ = 1930 A cm^−2^, which is four times higher than reference LEDs—by accelerating radiative recombination to outcompete Auger losses. Building upon the BRW architecture, Tan et al. pioneered a transformative circular Bragg resonator (CBR) platform [139]. The CBR achieves exceptional mode confinement (Γ = 89%) and a Purcell factor of 22.7, far exceeding those of conventional vertical-cavity surface-emitting lasers (VCSELs), enabling an ultralow lasing threshold of ~17 μJ cm^−2^ (70% lower than VCSELs). Moreover, its compact mode volume facilitates unprecedented integration density and exceptional operational stability. This CBR platform offers a new way to achieve electrically pumped CQD lasers.

#### 4.4.2. Photodetector

CQDs have become as a compelling platform for on-chip photodetectors, with recent advances addressing key challenges in spectral coverage, operational stability, and practical deployment through material and device innovations [140,141]. Stacked configurations enable broad visible-to-mid-wave infrared response [142], while in situ electric field-activated doping enhances large-format array uniformity via planar p-n junctions [143]. Heterojunction engineering extends operational temperatures and improves charge separation [144,145], and heavy-metal-free alternatives provide an ecofriendly solution without performance loss. Photonic integration (e.g., cavity-enhanced designs) enables precise spectral control, complementing broad response [146]. Interface optimization and avalanche mechanisms boost efficiency and gain, with stability improvements supporting practical use [147]. These advances position CQD detectors as versatile components for next-generation on-chip optoelectronics [22,148,149,150].

In 2024, Mu et al. demonstrated broadband imaging by employing a stacked lead sulfide/mercury telluride (PbS/HgTe) CQD configuration [141]. This architecture integrates visible PbS, short-wave infrared (SWIR) HgTe, and mid-wave infrared (MWIR) HgTe layers, enabling a broad spectral response (~0.4–5.0 μm). And the graded energy gap configuration facilitates directional carrier transport and minimizes recombination, which also contributes to the broad spectral response. This heterojunction strategy delivers remarkable array-scale uniformity: Focal plane arrays (FPAs) with 640 × 512 pixels exhibit pixel operability > 99.99%, supported by dark current mapping showing minimal variation and detectivity consistently exceeding 3.15 × 10^10^ Jones across the array. Further ex-tending the spectral capabilities of CQD photodetectors, Xue et al. demonstrated the successful photodetection of very long wave infrared (VLWIR) wavelengths up to 18 μm using large-size HgTe CQDs [150]. This breakthrough was achieved by employing a re-growth method and ionic doping modifications that significantly improve carrier mobility and stability.

**Figure 7:**
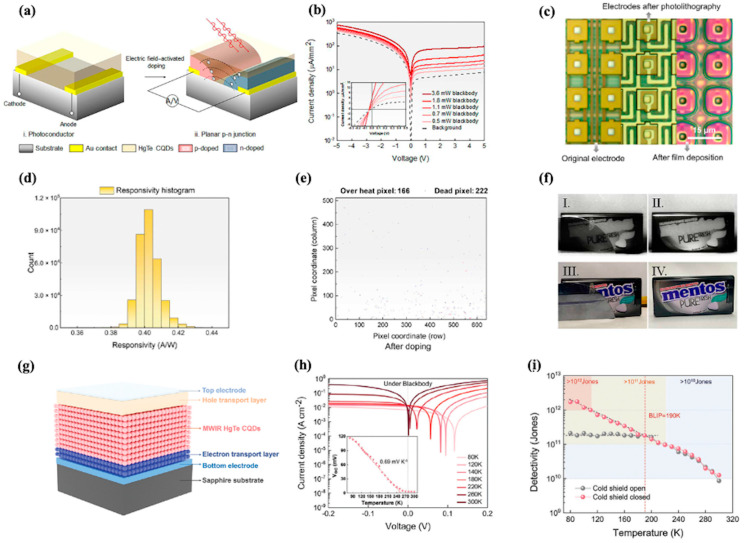
(**a**) Schematic illustrating the transition from photoconductive (PC) to photovoltaic (PV) operation via in situ electric field-activated doping. (**b**) Current density–voltage curves of HgTe CQD detectors with planar p-n junctions under dark and varying infrared intensities (blackbody). (**c**) Optical micrographs of electrodes for focal plane array (FPA) imagers before/after photolithography and after HgTe CQD film deposition, with an inset of the 640 × 512 pixel FPA chip. (**d**) Responsivity histogram of the planar p-n junction FPA imager. (**e**) Spatial distribution of noneffective pixels (overheated: 166; dead: 222) in the FPA imager after electric field-activated doping. (**f**) SWIR images (I, III) and visible images (II, IV) captured by the FPA imager, revealing details obscured in visible light [143]. (**g**) Schematic architecture of the MWIR CQD detector with band-engineered interfacial barriers. (**h**) Current–voltage curves of barrier heterojunction detectors under blackbody illumination at operating temperatures from 80 to 300 K. (**i**) Temperature-dependent detectivity of barrier heterojunction detectors, reaching background-limited infrared performance at 190 K (1.80 × 10^11^ Jones) and maintaining a high detectivity of 1.26 × 10^10^ Jones at room temperature (300 K) [145].

To further optimize the high-resolution SWIR infrared imaging, Qin et al. introduced an in situ electric-field-activated Cd^2+^-doping scheme to form lateral p–n junctions directly in HgTe CQD films (Figure 7a) [143]. This planar junction converts the device from a high–dark–current photoconductor into a zero-bias photovoltaic detector, driving dark currents down to ≈1 nA mm^−2^ at room temperature and yielding pronounced rectification in the I–V curves (Figure 7b). The 640 × 512 pixel focal plane array (FPA) exhibits exceptional uniformity, with root-mean-square roughness of HgTe CQD films as low as 5 nm (Figure 7c). Additionally, across a 640 × 512 pixel array, a tightly clustered responsivity of 0.40 ± 0.04 A W^−1^ (Figure 7d). Crucially, after doping with planar p-n junctions, overheat pixels decreased from 3716 to 166 and dead pixels dropped from 295 to 222 after doping (Figure 7e), resulting in an average D* ≈ 3 × 10^10^ Jones at λ = 2.5 µm. Figure 7f exhibits high-resolution SWIR images of objects hidden by a silicon wafer under visible light, illustrating that the in situ electric-field-activated planar p–n junctions enable room-temperature, zero-bias photovoltaic operation with array-scale uniformity.

Building on these advances in planar junction engineering, Mu et al. further pushed the operational boundaries of CQD photodetectors by developing band-engineered heterojunctions, enabling room-temperature MWIR detection and high-operation temperature thermal imaging [145]. As illustrated in Figure 7g, the device architecture integrates a ZnO electron transport layer, HgBr_2_-passivated HgTe CQD absorption layer, and P3HT/MoO_3_ hole transport layer, which forms interfacial barriers that suppress dark current while facilitating efficient photocarrier transport. This design yields pronounced rectification characteristics across a wide temperature range (80–300 K), with current–voltage curves under blackbody illumination revealing clear photovoltaic behavior and linearly decreasing open-circuit voltage with increasing temperature (Figure 7h). Critically, the heterojunction strategy enables exceptional detectivity performance as shown in Figure 7i. The detectors achieve a room-temperature detectivity of 1.26 × 10^10^ Jones, maintain background-limited infrared photodetection up to 190 K, and retain high sensitivity even at 250 K—addressing the long-standing challenge of cryogenic cooling in MWIR imaging.

Moreover, heavy-metal-free alternatives have emerged in recent years [151,152]. Wang et al. recently pioneered heavy-metal-free SWIR photodetection using phosphine-free Ag_2_Te QDs [153]. By introducing an AgBiS2 nanocrystal buffer layer to suppress interface recombination between SnO_2_ layer and Ag_2_Te layer, the device achieves a low dark current density of 6 μA cm^−2^ at −0.5 V and a room-temperature detectivity of 3 × 10^12^ Jones, >0.1 MHz bandwidth and >118 dB linear dynamic range over 350–1600 nm. Mao et al. fabricated silicon-based near-infrared photodetectors using EDT-modified Ag_2_Te QDs, achieving a high responsivity of 150 A W^−1^ and a specific detectivity of 4.8 × 10^12^ Jones at 1050 nm, with self-powered characteristics [154].

## 5. Conclusions

This review has systematically summarized recent advances in CQD-based multifunctional light-emitting devices and their on-chip integration. It highlights the synergies between material engineering, device design, and system-level innovation. From a fundamental perspective, the quantum confinement effect enables precise spectral control across the visible-to-infrared range. Meanwhile, carrier dynamics modulation via ligand engineering and core–shell heterostructures has unlocked high PLQY and suppressed nonradiative losses. This addresses critical bottlenecks in light emission efficiency. Synthetic methodologies, such as hot injection, LARP, and microfluidic flow synthesis, have advanced to the point that they can enable the large-scale production of high-quality CQDs with tailored sizes, morphologies, and surface chemistries. This lays the groundwork for the practical deployment of devices.

At the device level, CQD-based multifunctional systems have made significant strides in integrating light emission with information processing, energy harvesting, and memory storage. LEFETs have evolved from efficient emitters to multifunctional transceivers, such as light-emitting synaptic transistors, which can transmit photoelectric signals in parallel. LESCs have achieved power conversion efficiencies of over 15% and electroluminescence quantum efficiencies of 3.8% through rational ligand engineering. LEMs further expand the potential of CQD in neuromorphic computing by enabling light-modulated synaptic plasticity and optoelectronic neural networks with high-fidelity signal encoding. Meanwhile, on-chip integration has seen breakthroughs in CQD lasers, such as circular Bragg resonators with ultralow thresholds, and broadband detectors, such as PbS/HgTe stacks that cover 0.4–5.0 μm. These advancements address key limitations of traditional II–III–V/silicon heterointegration and pave the way for CMOS-compatible photonic circuits.

Despite these advancements, critical challenges remain. At material level, issues include the poor environmental stability of perovskite CQDs under moisture and thermal stress [155,156,157,158], persistent Auger recombination in small CQDs that limits electroluminescence efficiency at high current densities [159,160,161], and the need for heavy metal-free alternatives, such as InP/ZnS and Ag_2_Te, to balance performance with sustainability [162,163,164]. Device-level bottlenecks in LEFETs arise from carrier imbalance due to low hole mobility, trap-induced hysteresis, low external quantum efficiencies, and the fabrication complexity of ambipolar architectures, with Joule heating further limiting continuous brightness. In LESCs, intrinsic trade-offs persist. Enhancing emissive output often compromises photovoltaic performance, while interfacial nonradiative recombination, carrier imbalance, and ion migration undermine operational stability; lifetimes remain inadequate under sustained illumination and thermal stress. For LEMs, efficiency is hindered by Auger recombination which is intensified by Joule heating. Meanwhile the endurance and retention time of LEMs falls behind single-functional memristors, with cycling-induced degradation in switching uniformity and optical output. Stability under thermal, electrical, and environmental stress is further constrained by electrode diffusion, ion migration, and interfacial reactions. System-level integration also requires improved compatibility with existing CMOS processes to reduce contact resistance and minimize optical losses in on-chip photonic links.

Looking forward, breakthroughs in three interrelated areas will be pivotal. First, advanced material engineering, such as the atomic-layer deposition of passivating shells and in situ defect healing via dynamic ligands, will enhance stability and suppress nonradiative pathways. Second, innovative device architectures, such as 3D vertically stacked heterostructures and phase-separated mixed-dimensional junctions, will enable the unprecedented integration of light emission, energy conversion, and information processing. Third, data-driven design, leveraging machine learning to optimize synthesis parameters and device layouts, will accelerate the development of high-performance, scalable systems. These advances position CQDs to transform the optoelectronics landscape, enabling the development of compact, energy-efficient, multifunctional devices that bridge the gap between classical electronics and next-generation photonics.

## Figures and Tables

**Table 1:** Comparison of hot injection, LARP and microfluidic flow synthesis according to reaction conditions, advantages and disadvantages.

Methods	Conditions	Advantages	Disadvantages
Hot injection	- inert atmosphere - high temperature	- high crystallinity - high PLQY - adaptive to diverse material systems	- energy intensive - poor scalability - hightemperature side reactions
LARP	- ambient atmosphere - room temperature	- energy efficient - good scalability - no inertgas requirement	- low crystallinity - high surface defect density - broader size distribution
Microfluidic flow	- continuousflow microreactors - realtime optical monitoring	- high reproducibility and uniformity - finetuned reaction kinetics - MLdriven optimization	- operational complexity - high initial setup cost

## Data Availability

Data presented in this review are available on request from the corresponding authors.

## Abbreviations

The following abbreviations are used in this manuscript:

ASE	Amplified spontaneous emission
CQD	Colloidal quantum dot
DFB	Distributed feedback
EL	Electroluminescence/Electroluminescent
EQE	External quantum efficiency
ETL	Electron transport layer
HTL	Hole transport layer
LED	Light-emitting diode
LEFET	Light-emitting field-effect transistor
LEM	Light-emitting memristor
LESC	Light-emitting solar cell
PLQY	Photoluminescence quantum yield
PV	Photovoltaic
QD	Quantum dot
TRPL	Time-resolved photoluminescence
